# Supplementary figures and images for: From larva to adult: In vitro rearing protocol for honey bee (Apis mellifera) drones
Source: PLoS One. 2025 Feb 13;20(2):e0314859. doi: 10.1371/journal.pone.0314859 (PMC11824949; doi:10.1371/journal.pone.0314859)

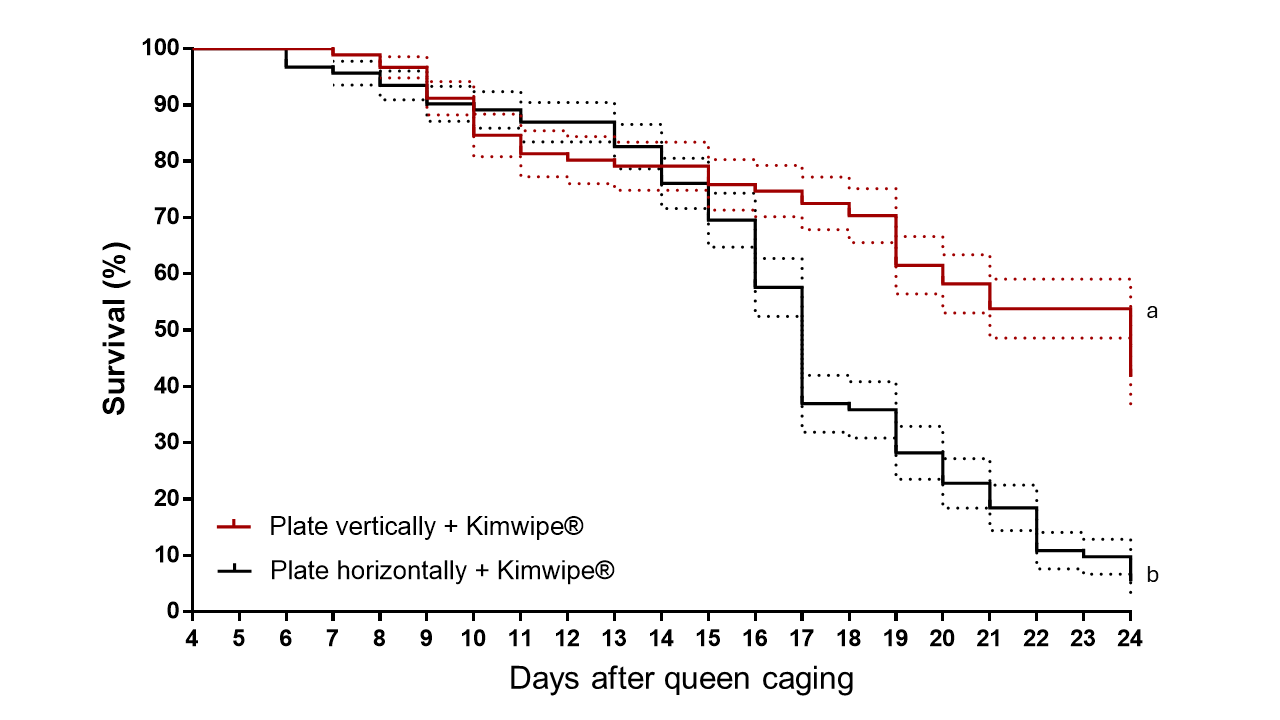

Supplement: S1 Fig — Percent survival (± SEM in dashed lines) over time in days (after queen caging) in either the horizontal plate orientation with Kimwipe® absorbent tissue (n = 92) or the vertical plate orientation with Kimwipe® (n = 91). Different letters signify statistical differences at α = 0.05. (TIF) [file pone.0314859.s001.tif]
